# Clinical evaluation of bond failures and survival between mandibular canine-to-canine retainers made of flexible spiral wire and fiber-reinforced composite

**DOI:** 10.4317/jced.51379

**Published:** 2014-04-01

**Authors:** Maria F. Sfondrini, Danilo Fraticelli, Linda Castellazzi, Andrea Scribante, Paola Gandini

**Affiliations:** 1 MD, DDS, PhD. Università degli Studi di Pavia - Dipartimento di Scienze Clinico-Chirurgiche, Diagnostiche e Pediatriche, Sezione di Odontoiatria, UDA di Ortognatodonzia e Odontoiatria Infantile. Italy; 2 MD, PhD. Università degli Studi di Pavia - Dipartimento di Scienze Clinico-Chirurgiche, Diagnostiche e Pediatriche, Sezione di Odontoiatria, UDA di Ortognatodonzia e Odontoiatria Infantile. Italy; 3 DDS. Università degli Studi di Pavia - Dipartimento di Scienze Clinico-Chirurgiche, Diagnostiche e Pediatriche, Sezione di Odontoiatria, UDA di Ortognatodonzia e Odontoiatria Infantile. Italy; 4 DDS, PhD. Università degli Studi di Pavia - Dipartimento di Scienze Clinico-Chirurgiche, Diagnostiche e Pediatriche, Sezione di Odontoiatria, UDA di Ortognatodonzia e Odontoiatria Infantile. Italy; 5 MD, DDS. Università degli Studi di Pavia - Dipartimento di Scienze Clinico-Chirurgiche, Diagnostiche e Pediatriche, Sezione di Odontoiatria, UDA di Ortognatodonzia e Odontoiatria Infantile. Italy

## Abstract

Objectives: The purpose of this longitudinal prospective randomized study was to evaluate the clinical reliability of two different types of postorthodontic treatment retainers: a silanised-treated glass fibers-reinforced resin composite (FRC) and a directly bonded multistranded stainless steel wire. The hypothesis of the study was to assess if significant differences are present between failure rates of the two retainers. Study Design: This prospective study was based on an assessment of 87 patients (35 men and 52 women),with an average age of 24 years who required a lower arch fixed retainer after orthodontic treatment. Patients were divided in two groups. Assignment was carried out with random tables. A follow-up examination was carried out once a month. The number, cause, and date of single bond adhesive failures were recorded for both retainers over 12 months. Teeth that were rebonded after failure were not included in the success analysis. Statistical analysis was performed by means of a Fisher’s exact test, Kaplan-Meier survival estimates, and log rank test. Results: Bond failure rate was significantly higher (P=0.0392) for multistranded metallic wire than for FRC. Conclusions: Glass fiber-reinforced resin composite retainers and multistranded metallic wires showed no significant difference in single bond failure rates over a one-year follow up.

** Key words:**Fiber reinforced composite, fixed retention, multistranded wire, orthodontics, retainer, splint.

## Introduction

In the last few years fiber-reinforced resin composite (FRC) has been introduced to dental practice. Embedding fibers (Polyethylene, Aramid, Carbon, Glass) into resin composite to reinforce the material properties is indicated for several clinical application, such as: periodontal tooth splinting, replacement of missing teeth, Maryland Bridge, complete denture repair, overdenture components, direct construction of posts and cores (1,2).

In orthodontics FRCs are used for active and passive applications, such as: increasing anchorage units and postorthodontic tooth retention (3). Fibers provided high mechanical properties (similar to metallic alloy), while resin composite offers good aesthetics benefits (4,5). Among advantages of fiber-reinforced resin composite can be considered its high biocompatibility (6): FRCs are metal-free and therefore indicated for patients allergic to metals or in subjects screened by Nuclear Magnetic Resonance (RMN). Another important property is aesthetics: fibers are barely invisible and don’t affect the teeth-translucence (7). This aspect is important, considering the higher number of adult patients who request an orthodontic therapy (8).

In vivo-vitro studies suggested that glass fiber-reinforced composite may be used for fixed lingual retention of the anterior segment after orthodontic treatment (3-8). Clinical reliability of conventional orthodontic retainers versus fiber reinforced composite splints has been tested (9). Moreover flexible spiral wire (10) and fiber reinforced composite (11) splints preparation has been described. In fact in literature many Authors evaluated both multistranded wires and fiber reinforced composites used for postorthodontic retention (12-19) showing detachment percentage after a 2-years follow up from 49 % to 91% (9,20). However only few controlled clinical follow-up studies based on comparison of a flexible spiral wire and a direct bonded glass fiber-reinforced resin composite are present.

The aim of this randomized study is to compare the clinical reliability of a resin composite retainer reinforced with glass fibers with a multistranded stainless steel wire, analyzing the number and the time of detachments during one year follow-up period.

## Material and Methods

The fiber tested in the present investigation is a FRC reinforced with silanised-treated glass fibers (Everstick Ortho, Stick Tech ltd, Turku, Finland). This fiber-reinforced retainer contained 1000 silanised glass fibers plunged in a monomer-polymer gel matrix. Fibers were pre-impregnated both with PMMA and with light-cured monomer (BIS-GMA) and they were covered with a thin layer of pre-impregnated PMMA. The retainer contains BIS-GMA, and no pre impregnation with light-cured resin is needed before clinical use (4,5,7).

In the present Randomized controlled trial 100 patients were selected. All patients were previously subjected to MBT fixed orthodontic treatment (mean duration: 22 months) and were enrolled for lower retention therapy from May 2009 to March 2011 in the Orthodontics Department of the University of Pavia, Italy. Data were recorded until March 2012 to have a minimum of 1 year follow up for all the patients. Patients with extraction cases, short time therapy and orthognatic surgery patients were excluded from the study.

Department Committee approved the study design. Principles outlined in the Declaration of Helsinki were followed. Written informed consent was obtained from all participating adult subjects and from parents or legal guardians for minors or incapacitated adults.

The retainer type was assigned to patients using randomization tables. 50 flexible spiral wire (Ortosmail, Krugg spa, Milan, Italy; Ø 0,0175”) and 50 FRCs splints (Everstick Ortho, Stick Tech ltd, Turku, Finland; Ø 0,5 mm) were bonded by one operator in the lower arch from right to left canine.

Both FRC and flexible spiral wires were applied according to the literature guidelines (10,14,19). The anterior dental arch segment was isolated with cheek retractors and rubber dam to control moisture contamination. The enamel was cleaned with sandblasting technique by particles of Al2O3 (Aluminium dioxide) after first cleaning by mixture of water and fluoride-free pumice using a rubber polishing cup in a low-speed handpiece. The teeth were rinsed with water and dried with an oil-free syringe and subsequently etched with 37% orthophosphoric acid gel (etching gel; 3M Unitek, Monrovia, Calif, USA) for 30 seconds. The acid was rinsed with water for at least 30 seconds and the enamel was thoroughly air-dried. The retainers were accurately located on the lingual surface and a thin layer of bonding Trasbond XT Primer (3M Unitek, Monrovia, Calif, USA) was applied and then light-cured with a halogen curing unit (Optilux 501; SDS Kerr, Danbury, Conn; light intensity, 930 mW/cm2; wavelenght range, 400-505 nm) for 20 seconds (19). A small account of Transbond XT Resin (3M Unitek, Monrovia, Calif, Usa) was placed to cover the retainer and light cured for 40 seconds a tooth, as suggested by the manufacturer. The splints were contoured to allow sufficient and easy cleaning of the interproximal areas (9,10). Finishing was conducted by diamonds burs and polishing discs. After bonding process each patient was instructed to appropriate oral hygiene technique.

All subjects were monthly recalled to evaluate possible detachments of the retainers from the teeth. Only first failure for each tooth has been considered. As in previous studies (17,19) all detachments happened at composite-enamel interface. No detachment were recorded at wire-composite or fiber-composite interface. The endpoint of the study was 12 months.

13 patients were dropped out from the study as they missed at least one control visit. Therefore a total of 87 patients (35 men and 52 women), with an average age of 24 years (14-62 years), completed the study. 47 flexible spiral wire and 40 FRCs splints were compared.

Statistical analysis was performed with a computer software (Stata 7, College Station, Tex) with the Fisher exact test. Because, in addition to the simple event of failure, it is interesting to consider the time that elapsed before bond failure, Kaplan-Meier estimates of survival curves were constructed and compared by using the log-rank test. The level of significance was set at *P* < 0.05.

## Results

At the end of the follow up the rates of detachments of the two different retainers were 17.73% (N=47) for flexible spiral wires and 11.25% (N=27) for glass fiber-reinforced resin retainers respectively; the data are shown in  Table 1. No significant difference in term of failure rates between the two different splints was detected (*P*>0.05).

**Table 1:**
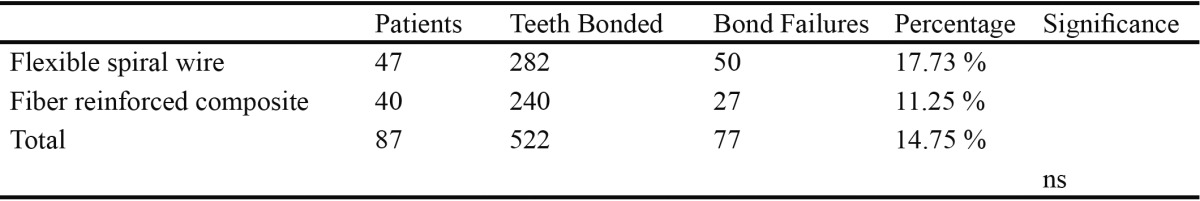
Distribution of bond failures for the different retainer types. Numbers of single tooth detachment.

**Figure 1:**
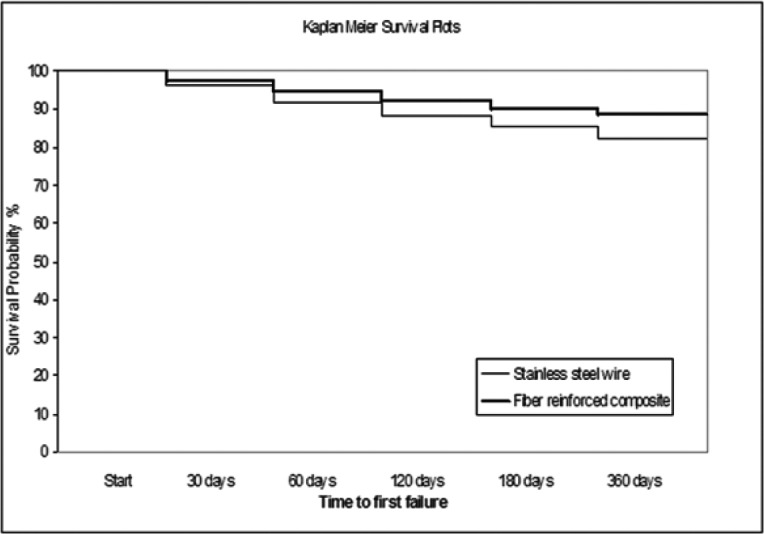
Kaplan Meier survival plots for stainless steel wire and FRC retainers.

Kaplan Meier survival plots for the two different types of retainers are showed in figure 1. No significant diffe-rence in retainer failure risk over the 12 months of follow up was found (Hazard Ratio: 1.64; Confidence Interval 95%: 1.02-2.54; log-rank test: *P*=0.0592).

## Discussion

The results of orthodontic treatment are potentially unstable, so permanent or semi-permanent retention with a fixed retainer is necessary (12-14). Multistranded flexible spiral wire retainers are widely accepted and they are considered the gold standard treatment option in modern orthodontics (5,10,20). Various studies tested the retentive efficiency and reliability of multistranded wire retainers bonded to lingual sides of canines and incisors (12-16).

Fiber-reinforced composites have been introduced in dental practice a few years ago and they are indicated for postorthodontic anterior teeth retention (3,6,11,12). Among the advantages of FRCs can be considered their high biocompatibility (7) (metal free material), bonding properties and aesthetics benefits (9). Different in vitro investigations carried out with FRCs made by silanised glass fibers showed that they had higher mechanical properties than unreinforced resin composite (17,18). values of flexural strength recorded with FRCs are demonstrated to be higher than stainless steel, similar to gold and Cr-Co alloy (4,17).

In this clinical prospective study, evaluating the one year follow up period, the number of FRC retainers which failed was not significantly different than for metallic wires. Kaplan Meier survival plots showed that clinical failure rate of FRC splints is unsignificantly lower than metallic retainers.

The clinical efficiency of FRC retainer system is probably based on the internal structure of the complex. The resin of the matrix and the adhesive system integrate with fibers. This homogeneous structure can allow mechanical stresses to be adsorbed and dissipated (4,5,17). In fact the external layer of PMMA, in contact with bonding agent, dissolved among the same adhesive to get into the groves created and to join the fibers physically. The internal matrix, instead, alloyed chemically with resin composite of the adhesive. Traditional retainer is a mechanical assembly between resin composite and stainless steel with a weakness point at the junction of the two materials, which are not able to bond chemically each other (4,5).

Despite several authors evaluated mechanical properties of FRCs (3,8,11), in literature there are few clinical investigations that compared failure rates of multistrand wire retainers with the ones of glass fiber-reinforced resin retainers (7,20). The reliability of posttreatment anterior segment retention with resin composite retainers reinforced with unidirectional glass fibers was compared with the reliability of multistranded wire retainer by Tacken *et al.* (20) This prospective study, demonstred that glass fiber retainers showed high failure rates in comparison with stainless steel wire retainers (51 versus 12 per cent). This result was in disagreement with present study, which indicated that glass fiber-composite splints were as reliable as multistranded metallic ones. The variability of results could be ascribed to the use of different materials and different bonding techniques. In fact a light emitting diode has been used to achieve polymerization, whereas in the present study a conventional light has been used. Moreover patients were monitored every 6 months, whereas in the present study patients were monthly recalled.

Bolla *et al.* (9), compared failure rates of the mandibular splints breakage rates over a 6 years follow up. These Authors found failure rate of 8.82% for the glass fiber-reinforced group and 15.62% for the multistranded stainless steel wire group. As in the present investigation no significant difference was reported between the 2 techniques.

In literature some investigations (1,19) tested polyethylene fiber reinforced composite (Ribbond) retainers found that fiber splints showed clinical reliability lower or comparable to metallic splints. Moreover in marginal areas polyethylene fibers may become exposed and come into contact with oral tissue, saliva and microbes and un-dergone occlusal forces (12,20-22). In fact, the reinforcement of polymers with a ribbon layer slightly increases the trasverse strength but the adherence of the polyethylene fibers to the base polymer have been shown to be poor (23). Moreover the retention of plaque (20-24) is another limitation. Bleeding on probing and bleeding intensity has been shown to be significantly increased over time in patients with FRC retainers when compared with patients with multistranded wires retainers (20). In fact also multistranded wire retention has been attested to show significantly higher bleeding scores in comparison with subjects without fixed retention, this indicating that both procedures could tamper with periodontal conditions (7,20).

Finally the difficulty to repair retainers when the bonding fails in one or more points (25) represent other limitations of both polyethylene and glass reinforced composite fibers.

A previous investigation evaluated different orthodontic adhesives for FRC bonding and Transbond XT (3M Unitek, Monrovia, Calif, USA) exhibited the higher shear bond strength values than both flowable composite and glass ionomer cement (26). Therefore the standardized procedure used in the present clinical study during FRC and metallic splints bonding is based on this adhesive system which is demonstrated to express higher bond strength than other adhesives tested (26).

When testing fixed orthodontic retention appliances, different authors (27) demonstrated that early failures are due to: (1) some degree of distortion during setting of adhesive, leading to a decrease in bond strength, (2) the use of too little adhesive, (3) direct trauma to the retainer. Moreover other studies showed that late failures may be associated with (1) composite abrasion, (2) low resistance to the fatigue or to excessive masticatory loads (28).

The results of the present clinical study showed no statistically significant differences in failure rates between multistranded wire retainers and glass fiber-reinforced resin composite ones over a one year follow up period. The present research indicated that both FRCs and flexible spiral wire retainers can be recommended for permanent lingual retention of the anterior segment after orthodontic treatment.

Reliability of fixed retention appliances is crucial to obtain long term stability of orthodontic results (29). In fact also etiologic factors of malocclusion should be determined at the time of the initial diagnosis and should be controlled during treatment and retention to prevent relapse.

Both techniques evaluated in the present investigation showed high success or failure rates. Limitations of the present investigations are related to the fact that only one type of fiber reinforced composite has been tested and to short time period of observation. Further studies with different fibers (30) or with repeated measures and further comparisons at 2 and 3 years of follow up would be an interesting prosecution of the present report.

## Conclusions

The present investigation showed that glass fiber-reinforced resin composite retainers and multistranded metallic wires showed no significant difference in single bond failure rates over a one-year follow up.
